# 379. Reduction in Cefepime Usage After Inclusion of SAAR Metric on the Inpatient Provider Scorecard

**DOI:** 10.1093/ofid/ofaf695.126

**Published:** 2026-01-11

**Authors:** Lauren McDaniel, Nicholas Stornelli, Thomas S Tuggle, Shyam Odeti, Kristina D Werner

**Affiliations:** Carilion Clinic, Roanoke, VA; Carilion Clinic, Roanoke, VA; Carilion Clinic, Roanoke, VA; Carilion Clinic, Roanoke, VA; Carilion Clinic, Roanoke, VA

## Abstract

**Background:**

The Joint Commission recommends antimicrobial stewardship programs (ASP) collect, analyze, and report data to hospital leadership and prescribers. Carilion Clinic regularly reported antibiotic usage (AU) data to leadership and quality departments but increasing visibility of AU metrics for frontline providers was a consistent challenge.

**Figure 1.**
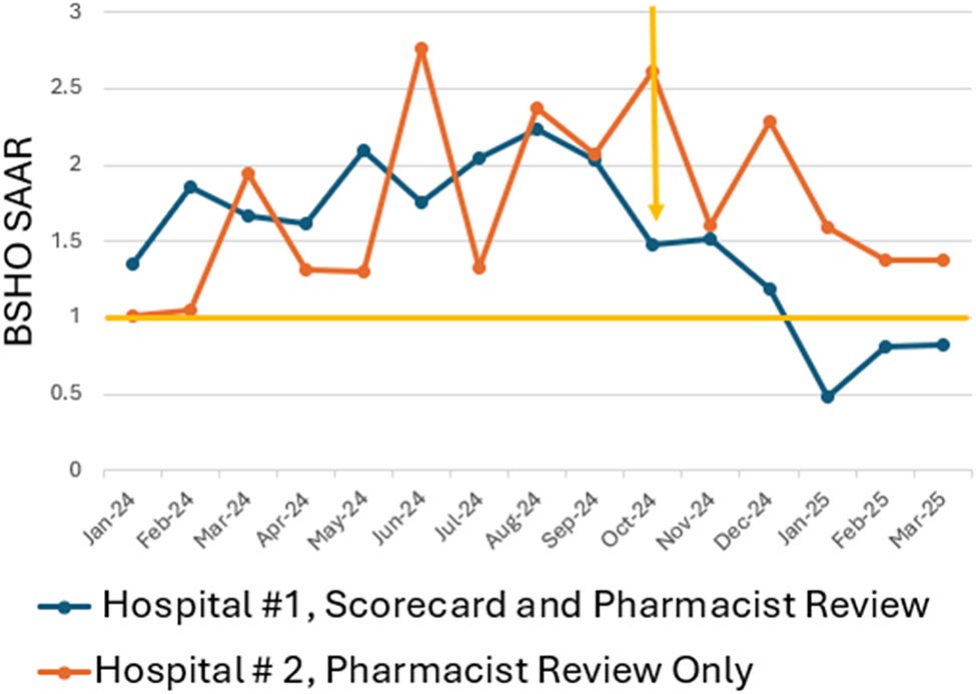
BSHO SAAR before and after implementation of scorecard and pharmacist PAF compared with control hospital with PAF but without scorecard implementation

**Methods:**

A quality improvement initiative was conducted with Carilion Clinic’s ASP and Hospital Medicine Section, incorporating a broad-spectrum antibacterial agents used for hospital-onset infections (BSHO) standardized antimicrobial administration ratio (SAAR) onto the hospitalist scorecard metric at a critical access hospital. The ASP team evaluated all SAARs within the previous 2 years and the BSHO SAAR consistently had higher observed AU compared with expected. The fiscal year (FY) of Oct-Sept 2023 BSHO SAAR was 1.64 and Oct-Sept 2024 was 1.80. An initial target of < 1.65 was selected to reduce BSHO SAAR by ∼10% from 2024. In addition to the scorecard metric, daily prospective audit and feedback (PAF) by ASP pharmacists started in October 2024 at all facilities. The goal of this study was to evaluate the difference in BSHO SAAR at critical access hospital 1 with PAF and the new scorecard measure compared with critical access hospital 2 which only had PAF.

**Results:**

After implementation of PAF, both hospitals saw a decrease in BSHO SAAR (Figure 1). Hospital 1, which had the scorecard measure in addition to PAF, saw a more significant decrease in BSHO SAAR. Cefepime is the main driver of the BSHO SAAR at both institutions (76%). At hospital 1, cefepime usage was 113 days of therapy DOT/1000 days present Oct 2023-Mar 2024 which decreased to 75 DOT/1000 days present after implementation Oct 2024-Mar 2025. Ceftriaxone, which is not included in the BSHO SAAR, was 140 DOT/1000 days present and after implementation it remained unchanged with 140 DOT/1000 days present.

**Conclusion:**

After pharmacist PAF both hospitals saw a reduction in the BSHO SAAR. Additionally, increasing provider awareness with a scorecard metric may have led to a reduction in utilization of cefepime while ceftriaxone not included on the scorecard remained unchanged.

**Disclosures:**

All Authors: No reported disclosures

